# DNA marker identification of downy mildew resistance locus Rpv10 in grapevine genotypes

**DOI:** 10.18699/VJGB-23-18

**Published:** 2023-04

**Authors:** E.T. Ilnitskaya, M.V. Makarkina, S.V. Toкmakov, L.G. Naumova

**Affiliations:** North-Caucasian Federal Scientific Center of Horticulture, Viticulture, Winemaking, Krasnodar, Russia; North-Caucasian Federal Scientific Center of Horticulture, Viticulture, Winemaking, Krasnodar, Russia; North-Caucasian Federal Scientific Center of Horticulture, Viticulture, Winemaking, Krasnodar, Russia; Ya.I. Potapenko All-Russian Research Institute of Viticulture and Winemaking – branch of Federal Rostov Agricultural Research Center, Novocherkassk, Russia

**Keywords:** Vitis sp., target alleles, Plasmopara viticola, DNA fingerprinting, Vitis sp., целевые аллели, Plasmopara viticola, ДНК-профилирование

## Abstract

One of the most common and harmful diseases of grapevine is downy mildew, caused by Plasmopara viticola. Cultivars of Vitis vinifera, the basis of high-quality viticulture, are mainly not resistant to downy mildew. Varieties with natural resistance to downy mildew belong to the vine species of North America and Asia (V. aestivalis, V. berlandieri, V. cinerea, V. labrusca, V. amurensis, etc.), as well as Muscadinia rotundifolia. The breeding of resistant cultivars is based on interspecific crossing. Currently, molecular genetic methods are increasingly used in pre-selection work and directly in breeding. One of the major loci of downy mildew resistance, Rpv10, was first identified in the variety Solaris and was originally inherited from wild V. amurensis. DNA markers that allow detecting Rpv10 in grapevine genotypes are known. We used PCR analysis to search for donors of resistance locus among 30 grape cultivars that, according to their pedigrees, could carry Rpv10. The work was performed using an automatic genetic analyzer, which allows obtaining high-precision data. Rpv10 locus allele, which determines resistance to the downy mildew pathogen, has been detected in 10 genotypes. Fingerprinting of grape cultivars with detected Rpv10 was performed at 6 reference SSR loci. DNA marker analysis revealed the presence of a resistance allele in the cultivar Korinka russkaya, which, according to publicly available data, is the offspring of the cultivar Zarya Severa and cannot carry Rpv10. Using the microsatellite loci polymorphism analysis and the data from VIVC database, it was found that Korinka russkaya is the progeny of the cultivar Severnyi, which is the donor of the resistance locus Rpv10. The pedigree of the grapevine cultivar Korinka russkaya was also clarified.

## Abstract (ru)

Милдью – одно из наиболее распространенных и вредоносных заболеваний виноградной лозы, возбудителем которого считают Plasmopara viticola. Сорта Vitis vinifera, выступая основой высококачественного виноградарства, практически не обладают генетической устойчивостью к милдью. Генотипы, имеющие природную устойчивость к поражению P. viticola, принадлежат видам винограда Северной Америки и Азии (V. aestivalis, V. berlandieri, V. cinerea, V. labrusca, V. amurensis и др.), а также к Muscadinia rotundifolia. По этой причине создание сортов винограда с повышенной устойчивостью к патогену основано на межвидовой гибридизации. В настоящее время молекулярно-генетические методы анализа все активнее используют на этапах предселекционной работы и непосредственно в селекции. Один из крупных локусов устойчивости к милдью – ген Rpv10 – впервые идентифицирован в сорте межвидового происхождения Солярис и изначально происходит от дикого амурского винограда. Известны ДНК-маркеры данного гена, позволяющие детектировать наличие Rpv10 в генотипах винограда. Методом ПЦР-анализа выполнен поиск доноров гена устойчивости среди генотипов 30 сортов винограда, которые, согласно родословным, могли бы нести ген Rpv10. Работа выполнена с использованием автоматического генетического анализатора, что позволяет получать высокоточные данные. По результатам ДНК-маркерного анализа в 10 генотипах винограда выявлено наличие аллели гена Rpv10, определяющей устойчивость к возбудителю милдью. Выполнено генотипирование сортов винограда, в которых обнаружен Rpv10, с помощью шести стандартных для ДНК-профилирования винограда SSR-маркеров. ДНК-маркерный анализ показал наличие аллели устойчивости у сорта Коринка русская, который, по общедоступным данным, является потомком сорта Заря Севера, не обладающим геном устойчивости Rpv10. С использованием анализа полиморфизма микросателлитных локусов и базы данных VIVC уточнена родословная сорта винограда Коринка русская. Установлено, что Коринка русская происходит от сорта Северный – донора локуса устойчивости Rpv10.

## Introduction

The Eurasian grapevine (Vitis vinifera L.) is the most widely cultivated and economically important fruit crop in the world (De Mattia et al., 2008). Grapevines are grown both for direct food consumption and for the production of wine. The issue of creating pathogen-resistant genotypes is relevant in the breeding of table and wine cultivars. Downy mildew is one of the most common and harmful diseases of grapevine, caused by biotrophic oomycete Plasmopara viticola Berl. et de Toni. The pathogen has a narrow specialization and affects only grapevines: it develops on all green organs of the plant – leaves, shoots, inflorescences, berries, tendrils. The greatest damage is caused to vineyards in warm periods with high humidity. The creation of new grapevine forms is based on the use of the genetic diversity. The searching and identification of genotypes – donors of resistance, is an important task both for studying the diversity of the existing gene pool and for the purposes of breeding new resistant cultivars.

The V. vinifera genotypes, being the basis of high-quality viticulture, are mainly not resistant to P. viticola. The breeding of resistant cultivars is based on interspecific crossing. Genotypes with natural resistance to downy mildew belong to the vine species of North America (V. riparia, V. aestivalis, V. berlandieri, V. cinerea, V. labrusca) and East Asia (V. amurensis, V. piasezkii), as well as Muscadinia rotundifolia (Alleweldt, Possingham, 1988; Wan et al., 2007). It is generally accepted that resistance in American species developed simultaneously with the pathogen, which is endemic to North America. Resistance to P. viticola in some forms of V. amurensis could have developed through evolution from resistance to P. cissii and P. amurensis, these microorganisms are endemic to Asia (Riaz et al., 2011).

Molecular genetic analysis methods are successfully used now to identify and map loci of resistance to downy mildew. Both major loci with large influence in phenotypic variation and minor loci with smaller effects were identified (Bellin et al., 2009; Di Gaspero et al., 2012; Schwander et al., 2012; Venuti et al., 2013; Ochssner et al., 2016; Divilov et al., 2018; Lin et al., 2019; Sapkota et al., 2019; Bhattarai et al., 2020; Sargolzaei et al., 2020; Fu et al., 2020). The results of many such studies are successfully used for DNA marker selection to create quality grape cultivars with pyramided resistance genes (Eibach et al., 2007; Zini et al., 2019; Possamai et al., 2020; Ruiz-García et al., 2021).

Thus, a major locus of resistance inherited from wild V. amurensis was identified in the genotype of interspecific cultivar Solaris, it was named Rpv10 (Schwander et al., 2012). The identified locus explained up to 50 % of observed phenotypic variance in the studied mapping hybrid population. Analysis of Solaris cultivar pedigree revealed that the allele that determines resistance to downy mildew was inherited from Severnyi (V. amurensis × Seyanets Malengra) cultivar. At the same time, studies have shown that in the genotype of Zarya Severa cultivar, which was selected from the same hybrid population as Severnyi (V. amurensis × Seyanets Malengra), the resistance allele is absent (Schwander et al., 2012). In the course of this study, flanking DNA markers of Rpv10 locus were identified, which make it possible to search for genotypes – donors of the downy mildew resistance locus Rpv10 in grapevine collections (Marker-Assisted Parental Selection) and in the breeding process to identify hybrid samples carrying the target allele (Marker-Assisted Seedling Selection) according to DNA analysis data.

The aim of the work was to determine Rpv10 locus in grape cultivar’s genotypes using flanking DNA markers.

## Materials and methods

Grapevine accessions and DNA extraction. We included in the study grape cultivars that could have the resistance locus Rpv10, according to analysis of their well-known pedigree: cultivars-descendants of Severnyi cultivar or bred using wild V. amurensis (original gene donor). In total, 30 genotypes were analyzed: Amurets, Avgusta, Buytur, Cabernet severnyi, Cvetochnyi, Denisovskiy, Dimatskun, Druzhba, Dushystyi, Fioletovyi ranniy, Golubok, Grushevskiy belyi, Korinka russkaya, Kostyukovskiy, Kristall, Kunleany, Kurchanskiy, Lusakert, Morozko, Murometc, Muscat donskoi, Pamyati Dombkovskoy, Saperavi severnyi, Skromnyi, Stanichnyi, Stepnyak, Sverkhranniy volgodonskiy, Vostorg, Vydvizhenets, Zolotoy Don cultivars. Plant material was collected from the Anapa ampelographic collection (North-Caucasian Federal Scientific Center of Horticulture, Viticulture, Winemaking) and the collection of Ya.I. Potapenko All-Russian Research Institute of Viticulture and Winemaking – branch of Federal Rostov Agricultural Research Center. Genomic DNA samples were isolated from young tops of plant shoots. DNA extraction was carried out by the method based on the use of CTAB (Rogers, Bendich, 1985).

DNA analysis. Three DNA markers were used to identify the allelic status of Rpv10 locus (GF09-44, GF09-46, GF09- 47). The sequence of primer oligonucleotides was synthesized according to information from the literature (Schwander et al., 2012). Polymerase chain reaction (PCR) was carried out in total volume of 25 μl containing about 50 ng of genomic DNA, 1.5 units of Taq-polymerase (SibEnzyme, Russia), 1X Taq-polymerase buffer (SibEnzyme, Russia), 2 μM of MgCl2 (SibEnzyme, Russia), 0.2 μM of each dNTP (SibEnzyme, Russia) and 200 μM of forward and reverse primers (Syntol, Russia). Amplification was carried out on a BioRad Thermo cycler T100 (USA). The following PCR conditions were used: initial denaturation for 5 min at 95 °C, 40 cycles of 30 s denaturation at 95 °C, annealing at 60 °C for 30 s and extension at 72 °C for 40 s, final step – 5 min extension at 72 °C. DNA of Solaris grape cultivar, which carries Rpv10 resistance allele, was used as a control to identify target alleles and correct the size of the detected PCR fragments

A standard set of SSR markers for DNA profiling of grapevine genotypes (VVS2, VVMD5, VVMD7, VVMD25, VVMD27, VVMD28, VVMD32, VrZAG62 and VrZAG79) was used for DNA fingerprinting of cultivars (This et al., 2004; This, 2007). Forward primers were labeled as follow: FAM (VVS2, VDMD27, VrZAG62), TAMRA (VVMD5, VVMD25, VVMD28, VVMD32), R6G (VVMD7, VrZAG79). The sequence of primer oligonucleotides was synthesized by Syntol (Russia). The following PCR conditions were applied: initial denaturation for 5 min at 95 °C; 34 cycles of 20 s denaturation at 95 °C, 30 s annealing at Tm (55 °C – VVS2, VVMD5, VVMD7, VVMD27; 58 °С – VrZAG62, VrZAG79; 60 °C – VVMD25, VVMD28, VVMD32) and 40 s extension at 72 °C; final extension of 3 min at 72 °C. To clarify the size of the detected alleles, we used the DNA of reference cultivars Cabernet Sauvignon and Pinot noir. Fragment analysis was carried out using an ABI Prism 3130 genetic analyzer. Molecular genetic studies were carried out using the instrument park of the center for collective use of technological equipment of the North Caucasian Federal Scientific Center for Horticulture, Viticulture, Winemaking

## Results and discussion

Rpv10 locus detection

At the first stage of the work, 30 grapevine accessions were analyzed using the GF09-46 marker, this microsatellite locus was identified as a closely linked DNA marker, correlating with the presence of Rpv10 locus, according to the studies of Schwander et al. (2012) (Schwander et al., 2012). The authors found that the PCR product of 416 base pairs size detected by the GF09-46 marker corresponds to the presence of Rpv10 locus allele which determines downy mildew resistance in the grapevine genotype. The target fragment was identified in ten cultivars on the 30 analyzed accessions: Augustа, Golubok, Denisovskiy, Dimatskun, Korinka russkaya, Morozko, Saperavisevernyi, Stanichnyi, Fioletovyi ranniy, Cvetochnyi (Table 1). Some of the results were published earlier (Ilnitskaya et al., 2019). At the second stage of the study, it was decided to analyze these ten cultivars with DNA markers GF09-44 and GF09-47, flanking the region of the chromosome where Rpv10 locus is localized, which makes it possible to make sure that there is no crossing-over at this locus in the studied genotypes (Schwander et al., 2012).

**Table 1.**
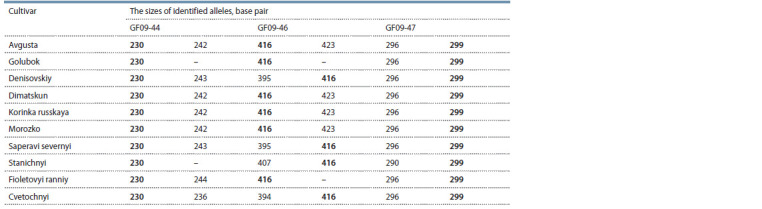
The results of grape genotypes analysis with DNA markers linked to downy mildew resistance locus Rpv10 Note. Target fragments that correlated with resistance are shown in bold.

Thus, according to the results of DNA marker analysis, target alleles at loci GF09-44 and GF09-47, correlating with the presence of a resistant allele in the Rpv10 locus, according to published data, were detected in all ten samples (see Table 1).

It has been determined that there was no crossing-over at the analyzed part of the chromosome in the studied genotypes, thus, according to the DNA marker analysis, the presence of the downy mildew resistance locus Rpv10 in grape cultivars Augustа, Golubok, Denisovskiy, Dimatskun, Korinka russkaya, Morozko, Saperavi severnyi, Stanichnyi, Cvetochnyi and Fioletovyi ranniy is confirmed.

An analysis of the pedigree of these cultivars suggests that the locus is inherited directly from Severnyi cultivar (Saperavi severnyi, Denisovskiy, Golubok, Fioletovyi ranniy, Cvetochnyi) and from the descendants of this cultivar (Avgusta, Morozko, Stanichnyi) (Table 2).

**Table 2.**
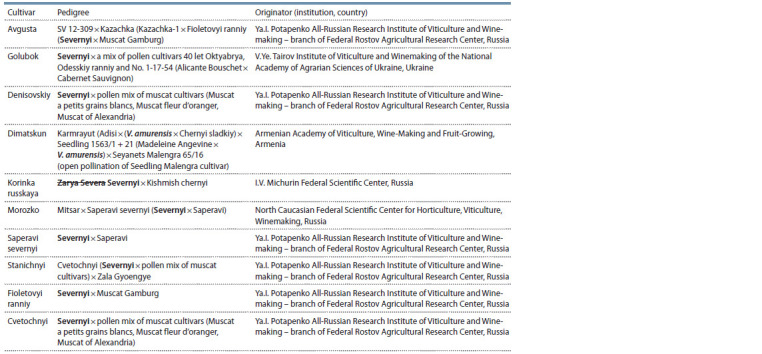
Pedigree of the analyzed grape genotypes

Fingerprinting

We carried out genotyping of Korinka russkaya and Zarya Severa by nine SSR loci used for DNAfingerprinting and identification of grapevine cultivars (This et al., 2004; This, 2007). The obtained data confirm the assumption that Zarya Severa cannot be the maternal parent of Korinka russkaya cultivar (Table 3).

**Table 3.**
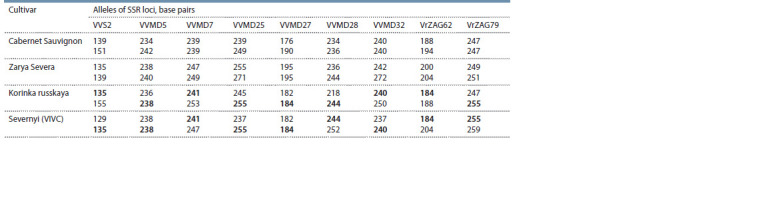
DNA profiles of grape cultivars Korinka russkaya, Zarya Severa and Severnyi by nine SSRs Note. The alleles inherited by Korinka russkaya from Severnyi genotype are shown in bold.

If Korinka russkaya was bred from Zarya Severa cultivar, then, according to the codominant type of inheritance of SSR loci alleles, one of the alleles of Zarya Severa of each analyzed microsatellite loci would be found in the corresponding locus of Korinka russkaya cultivar. However, in five (VVMD7, VVMD27, VrZAG62, VrZAG79, VVMD32) out of nine studied SSR loci, these cultivars do not have common alleles (see Table 3).

Most likely, Rpv10 locus in Korinka russkaya is inherited from Severnyi cultivar, according to the analysis of the history of Korinka russkaya genotype origin. In addition, the information that Severnyi cultivar is the parent of Korinka russkaya was found by us in a literary source describing the northern grape cultivars of Russia (Abuzov, 2009). Using data from the DNA profile database of Vitis International Variety Catalogue (http://www.vivc.de), we performed the DNA profiles comparison between Korinka russkaya and Severnyi. The allele from Severnyi cultivar was identified in each analyzed locus of Korinka russkaya, accordingly (see Table 3). So Severnyi is the parent of Korinka russkaya, Zarya Severa is not in the pedigree of Korinka russkaya.

We performed genotyping on VVS2, VVMD5, VVMD7, VVMD27, VrZAG62 and VrZAG79 SSR loci of cultivars, in which Rpv10 resistance locus was identified (Table 4). The DNA profiles can then be used for the trueness-to-type analysis of accessions. Genotypes Avgusta, Golubok, Denisovskiy, Dimatskun, Korinka russkaya, Morozko, Saperavi severnyi, Stanichnyi, Cvetochnyi and Fioletovyi ranniy can be used in breeding as donors of Rpv10. Аlso, аll these cultivars have increased frost resistance.

**Table 4.**
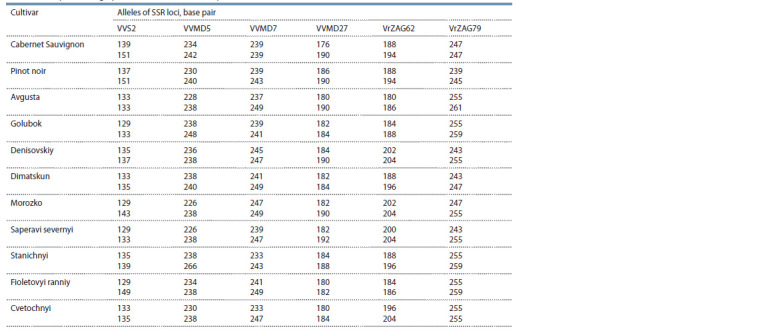
DNA profiles of grape cultivars with detected Rpv10 locus

## Conclusion

Using the DNA markers GF09-44, GF09-46 and GF09-47 linked to downy mildew resistance locus Rpv10, we analyzed 30 genotypes of grapes that could inherit this R-loci, according to their pedigrees. Rpv10 locus was detected in the DNA of cultivars Avgusta, Golubok, Denisovskiy, Dimatskun, Korinka russkaya, Morozko, Saperavi severnyi, Stanichnyi, Cvetochnyi and Fioletovyi ranniy. All these cultivars were genetically characterized with the standard set of six SSRs for identification of grape cultivars. It was also shown by the results of SSR analysis of Korinka russkaya and Zarya Severa genotypes that cultivar Zarya Severa is not the parent of Korinka russkaya. The presence of Rpv10 locus in Korinka russkaya genotype also confirms these data, since Zarya Severa does not carry Rpv10. Comparison of Korinka russkaya and Severnyi DNA profiles confirmed the assumption that Severnyi is the parent of Korinka russkaya cultivar. Thus, the pedigree of Korinka russkaya grape cultivar has been clarified.

## Conflict of interest

The authors declare no conflict of interest.
